# Pitaya allergy: a case report of anaphylaxis in a patient without cross-reactive allergens

**DOI:** 10.1186/s13223-025-00962-7

**Published:** 2025-05-05

**Authors:** Hannah Martin, Peter Stepaniuk

**Affiliations:** 1 Faculty of Medicine, University of British Columbia, Vancouver, BC Canada; 2 Division of Allergy and Immunology, University of British Columbia, Room 166– 1081 Burrard Street, Vancouver, BC Canada

**Keywords:** Pollen food allergy syndrome, Anaphylaxis, Pitaya, Cross-sensitization, Dragon fruit

## Abstract

**Background:**

Pitaya, commonly known as dragon fruit, is increasingly available and has allergenic potential. Pollens have been found to have cross-reactivity and thus induce allergies to several fruits, however, to our knowledge this is first report of pitaya anaphylaxis in a patient without co-sensitization to other fruit or environmental allergens.

**Case presentation:**

A 26-year-old male presented to the emergency department with anaphylaxis after consumption of pitaya (dragon fruit). He had no prior history of atopy. Epicutaneous skin testing demonstrated positive to pitaya and negative to all other cross-reactive food and environmental allergens, suggesting his pitaya allergy did not derive from cross-sensitization.

**Conclusions:**

Our case is unique in demonstrating the potential for pitaya allergy to occur independent of other allergies and cross-sensitization. Future research is warranted into suspected allergenic proteins in pitaya and quantifying their structural similarity to other known allergens.

## Background

Increasing globalization of food supply brings greater accessibility of a wider range of food products and potential allergens. One such food, known as dragon fruit or pitaya, is a member of the *Cactaceae* family, native to Guatemala, Costa Rica, El Salvador and Mexico [1]. Pitaya consumption can prompt allergic reactions and anaphylaxis and Hao et al. identified the suspected allergenic proteins to be cupin_1, HSP sti1-like and HSP70 in red-fleshed pitaya seeds and cupin_1 and HSP70 in white-fleshed pitaya seeds [2]. Hao et al. attempted to identify those allergenic proteins most similar to the pitaya seed allergenic proteins and their likely sources (Table 1). Pitaya flesh may also contain an allergenic protein, with the lipid transfer protein identified as a potential culprit [3].

There is limited published data on reported allergic reactions to pitaya. One prior report describes an allergic reaction without systemic symptoms prompted by the ingestion of red pitaya fruit with no mention of co-sensitization [4]. A second report detailed an anaphylactic reaction to pitaya postulated to be due to cross-sensitization to pollen-related lipid transfer proteins from an established birch pollen or mite allergy [3]. There exists other research similarly investigating the cross-reactivity connection between pollen and various fruits; the suspected allergenic proteins in pitaya are similar in structure to those of pollen and various dust mites [2, 5]. Our report describes pitaya-induced anaphylaxis in a patient lacking pollen, dust mite, coconut and/or latex cross-sensitization.

**Table 1:** The predicted similar allergenic proteins to the Pitaya allergenic proteins and their likely sources, adapted from Hao et al. [2]

Allergenic protein in pitaya	Predicted similar allergenic protein	Predicted source of similar allergenic protein
Cupin_1	Coc n1	Coconut
HSP sti1-like	Hev b5	Latex
HSP70	Tyr p28	Storage mites

## Case presentation

A healthy 26-year-old male living in North Vancouver, British Columbia, Canada presented to the emergency department with a history of sudden onset nausea, diarrhea, abdominal cramping, urticaria, periorbital angioedema, dysphagia and dyspnea. His symptoms began 15 min after he consumed a homemade smoothie containing cream of coconut, mango, banana, passion fruit and red pitaya. His initial vitals when presenting to the emergency department were blood pressure 129/68, a regular heart rate at 78 bpm, 18 breaths/minute and SpO2 98% with a temperature of 36.9˚C. Physical exam by the emergency room physician was notable for diffuse urticaria and increased work of breathing. He was diagnosed with anaphylaxis and treated with oral diphenhydramine, 0.3 mg intramuscular epinephrine, oral loratadine and oral prednisone. Symptoms resolved within 20 min of epinephrine administration. The patient denied any infectious symptoms on the day of his reaction. There were no co-factors (i.e. exercise, NSAID ingestion, fever, alcohol) reported by the patient.

He had no prior history of anaphylaxis, food allergy, asthma, rhinitis or atopic dermatitis. He was subsequently evaluated as an outpatient and at the time of initial consultation, he had been avoiding all components of the smoothie. Epicutaneous skin testing was performed with commercial extracts for environmental allergens and to coconut. These commercial extracts were obtained from Stallergenes Greer. Fresh food prick-by-prick skin testing was performed for coconut, kiwi, pitaya and mango. The patient had positive skin testing to fresh pitaya at 6 mm. The fresh food prick-by-prick skin test for pitaya was performed on a healthy volunteer and was negative, decreasing the likelihood of the patient’s result being a false positive. The patient had no reported history of reactions to latex after several prior exposures, and epicutaneous testing for latex was not done.

Given the possibility of pollen food allergy syndrome, he also underwent environmental testing. Epicutaneous skin testing was negative for dust mite (*D. farinae*,* D. pteronyssinus*), cat, dog, grass pollens (including timothy, orchard and rye), various tree pollens (including alder and birch), weed pollens (including ragweed) and various molds. Histamine and saline controls were appropriate. These findings suggested that the pitaya caused his index reaction. The patient was counselled to strictly avoid pitaya and he was advised that he could re-introduce all other fruits back into his diet. The patient was re-assessed about 1 year after his index reaction and had not had any further systemic reactions. He had continued to strictly avoid pitaya, but had re-introduced coconut, mango, banana, passion fruit back into his diet which he tolerated with no symptoms. He had also had ongoing periodic exposure to latex with no reaction.

## Discussion and conclusions

Based on our research, this is the first case report of a patient with anaphylaxis to pitaya in the absence of co-sensitization to pollens. Birch pollen (Bet v 1) is heavily implicated in pollen food allergy syndrome (PFAS) and prompts cross-sensitization to various vegetables, nuts, and fruits in up to 70% of patients with a birch pollen allergy [6, 7]. Birch pollen and its associated PFAS typically involves members of the fruit Rosaceae family (cherry, peach, pear, apples), nuts (hazelnut) and vegetables of the *Apiaceae* family (celery and carrot) [6]. While pitaya does not belong to any of these families, a previously published case report of pitaya anaphylaxis by Kleinheinz et al. postulated that a pollen-related LTP could have prompted cross-sensitization with pitaya in their patient [3]. After measuring the IgE reactivity of the patient’s serum to pitaya juice, they identified a non-specific lipid transfer protein (nsLTP) as the likely culprit allergen in their western blot. Their patient had never eaten pitaya before and they hypothesized that their patient’s sensitization occurred through a pollen-related LTP. However, this hypothesis seems less likely in our patient as there was no evidence of pollen sensitization.

Hao et al. predicted some potentially similar allergenic proteins to those of pitaya could be found in coconut tree pollen (Coc n 1), latex (Hev b 5) and storage mites (Tyr p 28), but our patient did not have sensitization to any of these allergens [2]. To date there are no publications confirming or quantifying similarities between pitaya allergenic proteins and similar allergens predicted by Hao et al. This too may be due to pitaya allergy being relatively uncommon. Our case report establishes that pitaya allergy can develop independent of other allergies.

Unfortunately, we did not have the facilities to perform in vitro analysis to identify the culprit allergen in our patient, which represents a potential avenue for future research. Additional research is warranted to determine which of the suspected allergenic proteins of pitaya (cupin_1, HSP sti1-like, HSP70) are more likely to lead to systemic reactions in patients who are mono-sensitized to pitaya.

## Data Availability

No datasets were generated or analysed during the current study.

## Abbreviations

PFAS: Pollen food allergy syndrome
LTP: Lipid transfer protein
HSP: Heat stable protein
